# Clinical and Virological Characteristics of Chronic Hepatitis B Patients with Coexistence of HBsAg and Anti-HBs

**DOI:** 10.1371/journal.pone.0146980

**Published:** 2016-01-11

**Authors:** Yong Liu, Le Zhang, Jin-Yong Zhou, Jinshun Pan, Wei Hu, Yi-Hua Zhou

**Affiliations:** 1 Department of Experimental Medicine, Nanjing Drum Tower Hospital, Nanjing University Medical School, Nanjing, 210008, China; 2 Jiangsu Key Laboratory for Molecular Medicine, Nanjing University Medical School, Nanjing, 210008, China; 3 Department of Infectious Diseases, Nanjing Drum Tower Hospital, Nanjing University Medical School, Nanjing, 210008, China; University of Cincinnati College of Medicine, UNITED STATES

## Abstract

Coexistence of hepatitis B surface antigen (HBsAg) and antibody against HBsAg (anti-HBs) comprises an atypical serological profile in patients with chronic hepatitis B virus (HBV) infection. In this study, in total 94 patients with coexisting HBsAg and anti-HBs and 94 age- and sex-matched patients with positive HBsAg were characterized by quantitatively measuring HBsAg and HBV DNA, sequencing large S genes, and observing clinical features. Compared with common hepatitis B patients, the patients with coexisting HBsAg and anti-HBs had lower HBsAg and HBV DNA levels. These two groups had similar rate of pre-S deletion mutations. However, in patients with coexisting HBsAg and anti-HBs, more amino acid substitutions in the *a* determinant of S gene were observed in HBV genotype C, but not in genotype B. Fourteen patients with coexisting HBsAg and anti-HBs were followed up for an average of 15.5 months. There were no significant changes in the levels of HBsAg, anti-HBs, HBV DNA and ALT over the follow-up period. Compared with the baseline sequences, amino acid substitutions in the MHR of HBsAg occurred in 14.3% (2/14) patients. In conclusion, coexistence of HBsAg and anti-HBs may be associated with higher frequency of mutations in the *a* determinant of HBV genotype C.

## Introduction

Hepatitis B virus (HBV) infection is a global health problem and common cause of liver cirrhosis and hepatocellular carcinoma (HCC). The natural course of HBV infection is a dynamic interplay of complex interactions between virus replication and host immune response [1]. Hepatitis B surface antigen (HBsAg) is the earliest seromarker of HBV infection [2]. If the host is able to clear the virus, HBsAg will become undetectable and antibody against HBsAg (anti-HBs) will develop. The emergence of anti-HBs following HBV infection generally indicates recovery and immunity to HBV infection [3].

Despite the aforementioned dynamics, coexistence of HBsAg and anti-HBs in chronic HBV-infected patients has been reported [4–6]. The rate of coexisting HBsAg and anti-HBs in chronic HBsAg carriers was estimated to be 2.43–8.9% [7–11]. However, the clinical and virological characteristics of these patients are not well described. Several reports showed that HBV mutations, which might be attributed to the selection of immune escape mutations, could explain the coexistence of HBsAg and anti-HBs in HBV infected patients [6,8,11–14]. In this study, we investigated the clinical and virological characteristics of the patients with coexisting HBsAg and anti-HBs. Furthermore, the mutations of HBV in these patients, including pre-S deletion mutations, S and reverse transcriptase (RT) mutations, were analyzed.

## Materials and Methods

### Study subjects

During January 2008 to April 2010, in total 13080 patients were defined with HBV infection based on the presence of HBsAg, which was detected by commercially available enzyme-linked immunosorbent assay (ELISA) kits (Huakang Biotech, Shenzhen, China) or microparticle enzyme immunoassay (Architect system, Abbott, North Chicago) in Department of Laboratory Medicine, Nanjing Drum Tower Hospital. Participants were included in the study if: 1) subjects had coexisting HBsAg and anti-HBs, 2) at least 1 ml fresh blood samples were available for further analysis, 3) patients were not infected with hepatitis C virus, hepatitis D virus or human immunodeficiency virus, 4) individuals gave the signed informed consent to use the samples in this study. In total 94 patients were assigned to the research group. In addition, 94 age- and gender-matched chronic HBV-infected patients with negative anti-HBs were also enrolled as a control group. To avoid cross-contamination, the serum samples were directly obtained by venipuncture from the patients or had been only used to detect HBV DNA in the routine laboratory tests, thus, all the samples had not been contaminated by other individuals’ samples. This study was approved by the institutional review boards (IRB) of Nanjing Drum Tower Hospital, in accordance with the Helsinki Declaration. In this study, each subjects signed the written informed consent.

### Detection of serological markers for HBV infection

All the 188 serum samples from the two groups were quantitatively tested for HBsAg and anti-HBs with microparticle enzyme immunoassay. The HBsAg > 0.05 IU/ml was defined to be positive, and anti-HBs ≥10 mIU/ml was defined to be positive. Hepatitis B e antigen (HBeAg), antibody against HBeAg (anti-HBe) and antibody against hepatitis B core antigen (anti-HBc) were also detected by commercially available ELISA kits or microparticle enzyme immunoassay.

### Quantitative assay of HBV DNA

The concentration of HBV DNA in serum samples was quantified by real-time PCR with a commercially available fluorescent real-time PCR assay (Shenyou Biotechnology, Shanghai, China), which has an internal quality control prepared by Clinical Laboratory Center of Chinese Ministry of Health. The lower detection limit of this assay was 100 IU/ml with a linear range of up to 10^8^ IU /ml.

### HBV DNA sequencing

The DNA was extracted from 200 μl serum with proteinase K digestion and phenol-chloroform extraction methods and dissolved in 20 μl Tris-EDTA buffer. Nested PCR was performed using two sets primers derived from the pre-S1 through S genes of HBV to amplify the sequences of the large S gene as described previously [15,16]. The external primer pairs were 5'-ATGYAGTTAATCATTACTT-3' (sense, nucleotide 2713–2731) and 5'-CCTTGATAGTCCAGAAGAAC-3' (antisense, nucleotide 459–440), and 5'-YTGGCCAAAATTCGCAGTC-3' (sense, nucleotide 300–318) and 5'-AAACCCCARRAGACCCACAA-3' (antisense, nucleotide 1017–998), respectively. The internal primer pairs were 5'-ACACGYAGCGCCTCATTTT-3' (sense, nucleotide 2793–2811) and 5'-GAAGATGAGGCATAGCAGCAGG-3' (antisense, nucleotide 433–412), and 5'-CTCCARTCACTCACCAAC-3' (sense, nucleotide 325–342) and 5'-TGACAKACYTTCCAATCAAT-3' (antisense, nucleotide 992–973), respectively. The PCR products were purified and sequenced directly on an ABI Prism 3130 sequencer (Applied Biosystems, Hitachi, Tokyo, Japan) after reaction with BigDye Terminator v3.1 (Applied Biosystems, Foster, CA). Genotype analysis was carried out on the obtained sequences and compared with the HBV S sequences of different HBV genotypes from the NCBI website (URL: http://www.ncbi.nlm.nih.gov/projects/genotyping/view.cgi?db=2). The phylogenetic tree was constructed based on gene sequences of HBV S region using Megalign sequence analysis software, Lasergene (DNASTAR, Madison, WI). Meanwhile the HBV mutations were identified through comparison the sequences with known wild-type virus from GenBank (genotype B: AF100309, AY163869 and DQ377158, genotype C: AF411409, AY040627 and DQ922651).

### Follow-up

Fourteen patients with HBsAg- and anti-HBs-positive were followed up for an average of 15.5 months (range 4–33 months) in this study. At the end of follow-up, the serum samples were collected, and the serological markers were tested. The HBV DNA was quantified, and the large S gene was amplified by nested PCR and sequenced.

### Statistical analysis

All the HBV DNA levels below the detection limit (100 IU/ml) were arbitrarily estimated as 1 log10 IU/ml in analysis. Statistical analysis was performed with Pearson’s Chi-square test, Student’s t test and Mann-Whitney U test where appropriate. All estimates were accompanied by two-sided P values of less than 0.05 were considered statistically significant.

## Results

### General characteristics of the study population

In total 13080 patients were positive for HBsAg, of which 436 (3.33%) had coexisting HBsAg and anti-HBs, and 94 were selected as research group in the current study. They were 56.8 ± 15.9 years old (range, 21–84), and 58 (61.7%) were male (Table 1, S1 Table). The patients in the control group were 55.1 ± 12.8 years old (range, 20–83), and 60 (63.8%) were male. The median serum ALT level of the patients was 23.6 (15.9, 47.9) U/L in the research group and 46.9 (24.6, 132) U/L in the control group (P < 0.01).

**Table 1:** General characteristics of patients with coexisting HBsAg and anti-HBs and controls.

	Patients with coexisting HBsAg and anti-HBs (n = 94)	Control group (n = 94)	P value
**Age**	56.8 ± 15.9	55.1 ± 12.8	0.493
**Gender (M/F)**	58/36	60/34	0.763
**HBsAg**			
** > 250 IU/ml**	25/94 (26.6%)	57/94 (60.6%)	< 0.01
**HBV DNA (Log10 IU/ml)**	3.36 ± 1.69	4.15 ± 1.55	0.001
**HBeAg positive**	30/94 (31.9%)	36/94 (38.3%)	0.359
**ALT**	23.6 (15.9, 47.9)	46.9 (24.6, 132)	< 0.01
**HBV Genotype**			
** B**	23/64 (35.9%)	24/71 (33.8%)	0.795
** C**	41/64 (64.1%)	46/71 (64.8%)	0.930
** D**	0	1/71 (1.4%)	

### Virological characteristics of HBV in patients with coexisting HBsAg and anti-HBs

Since coexistence of HBsAg and anti-HBs comprises an unusual serological pattern, we compared the virological characteristics of HBV infection between these two groups. As shown in Table 1, the proportion of patients with HBsAg level > 250 IU/ml in the research group was significantly lower than that in the control group (27.3% vs. 73.6%, P < 0.01). The HBV DNA levels in the research group were lower than that in the control group (3.36 ± 1.69 vs. 4.15 ± 1.55 log10 IU/ml, P = 0.001), and the proportion of patients with HBV DNA level > 10^3^ IU/ml in the research group was also significantly lower than that in the control group (67.2% vs. 87.7%, P = 0.008). Among the 94 patients with positive anti-HBs, the large S gene was successfully amplified by PCR and sequenced in 64 patients. The Phylogenetic tree showed that 23 (35.9%) were HBV genotype B, and 41 (64.1%) were genotype C (S1 Fig). Among the 94 patients in control group, the large S gene was successfully amplified and sequenced in 71 patients, in whom 24 (33.8%) were infected with genotype B, 46 (64.8%) were infected with genotype C, and one (1.4%) was infected with genotype D. There was no significant difference in genotypes between the two groups (genotype B: 35.9% vs. 33.8%, P = 0.80, genotype C: 64.1% vs. 64.8%, P = 0.93). Since the virus in each patient with positive anti-HBs was genotype B or C, we just analyzed the 70 patients with genotype B or C in the control group.

### Pre-S deletion mutations

Mutations in pre-S genes of HBV may represent an immune escape mutation since the regions contain several epitopes for T or B cells [17]. Furthermore, it was reported that pre-S deletion may decrease the expression of HBsAg, resulting in a low concentration of extracellular S gene products in the blood [18]. Of the 64 subjects with coexistence of HBsAg and anti-HBs, 11 samples (17.2%) harbored pre-S deletion mutations (Table 2), while the rate of pre-S deletion mutations was 18.6% (13/70) in the control group (P = 0.835).

**Table 2:** Comparison the amino acid mutations of HBV S gene between the patients with coexisting HBsAg and anti-HBs and controls.

	Patients with coexisting HBsAg and anti-HBs (n = 64)	Control group (n = 70)	P value
**S region**			
** N-terminal region (aa 1–99)**	3.11%	3.00%	0.72
** MHR (aa 100–169)**	2.67%	1.94%	0.017
** *a* determinant (aa 124–147)**	4.36%	2.32%	< 0.01
** C-terminal region (aa 170–226)**	2.93%	1.68%	< 0.01
**Complete HBsAg**	2.96%	2.34%	0.001
**Complete RT**	1.82%	1.43%	0.007
**Pre S1 (pol deletion)**			
** ATG**	2/64	2/70	0.927
** aa deletion**	4/64	4/70	0.896
**Pre S2**			
** ATG**	9/64	10/70	0.97
** aa deletion**	11/64	13/70	0.835

### Mutations in the S gene

**Fig 1:**
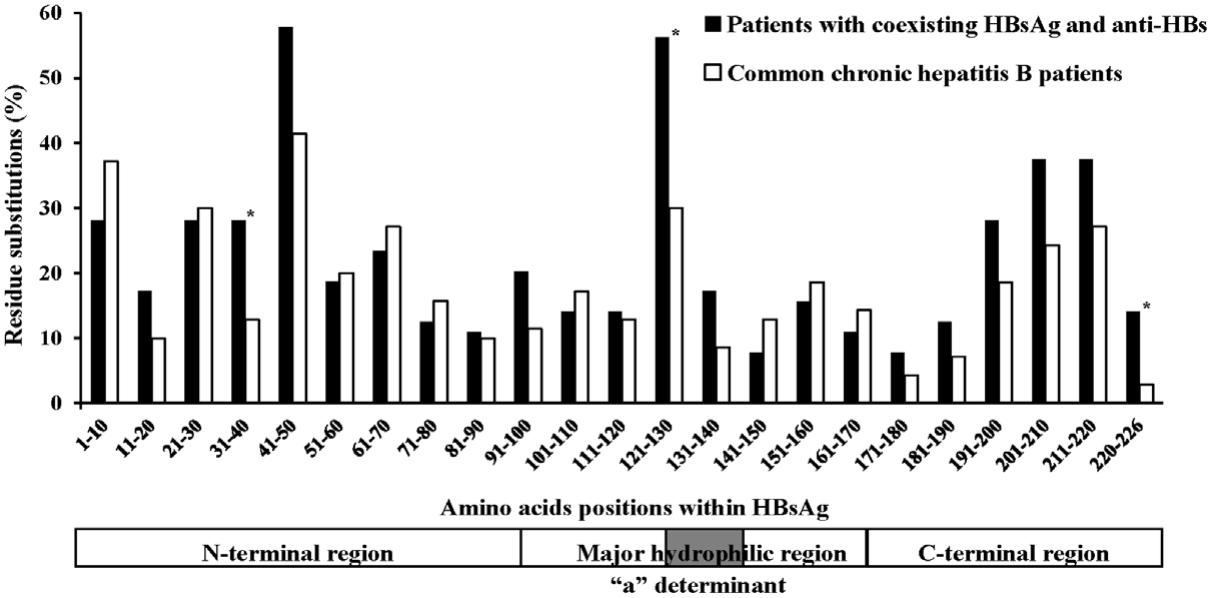
Amino acid mutation within HBsAg. Amino acid substitutions from chronic HBV-infected patients with or without anti-HBs positive in serum (analyzed at intervals of 10 amino acids) were identified through comparison the sequences with known wild-type virus from GenBank. Each bar represents the percentage of patients with mutated amino acid residues in each group at each interval of 10 amino acids per group. *Indicating the subregion harboring sequences mutations in patients with coexisting HBsAg and anti-HBs was significantly more than that in the control patients.

HBsAg sequences obtained from 64 patients with coexisting HBsAg and anti-HBs were compared with those from 70 chronic HBV-infected patients without anti-HBs. HBsAg is divided into three different subregions: the N-terminal region (amino acid 1 to 99), the major hydrophilic region (MHR, amino acid 100 to 169), which contains the ‘‘a” determinant, and the C-terminal region (amino acid 170 to 226). The amino acid variability within the S gene from patients in the research group was higher than that from control group (mean number of substitutions per 100 amino acid of 2.96 vs. 2.34, respectively, P = 0.001) (Table 2, Fig 1). Similarly, the amino acids variability in the C-terminal region and MHR was higher from patients in the research group than that from control group (mean number of substitutions per 100 amino acid of 2.93 vs. 1.68 in the C-terminal region, 2.67 vs. 1.94 in the MHR, respectively, P < 0.05) (Table 2). Meanwhile, an average rate of mutation in the ‘‘a” determinant of patients from the research group was 4.36 per 100 amino acid residues, higher than that in the patients from control group (2.32 per 100 amino acid residues, P < 0.01). In contrast, there was no significant difference in the amino acids variability of the N-terminal region between the two groups (mean number of substitutions per 100 amino acid of 3.11 vs. 3.00, P = 0.72).

### Mutations in HBV RT region

The RT region overlaps HBsAg at RT amino acid 8–236, with the HBsAg open reading frame (ORF) shifted downstream by 1 nucleotide. We also analyzed the RT subregion that overlaps HBsAg. The amino acid variability within the RT region from patients in the research group was higher than that from the patients of control group (mean number of substitutions per 100 amino acid of 1.82 vs. 1.43, respectively, P = 0.007) (Table 2).

### Point mutations in the *a* determinant

**Fig 2:**
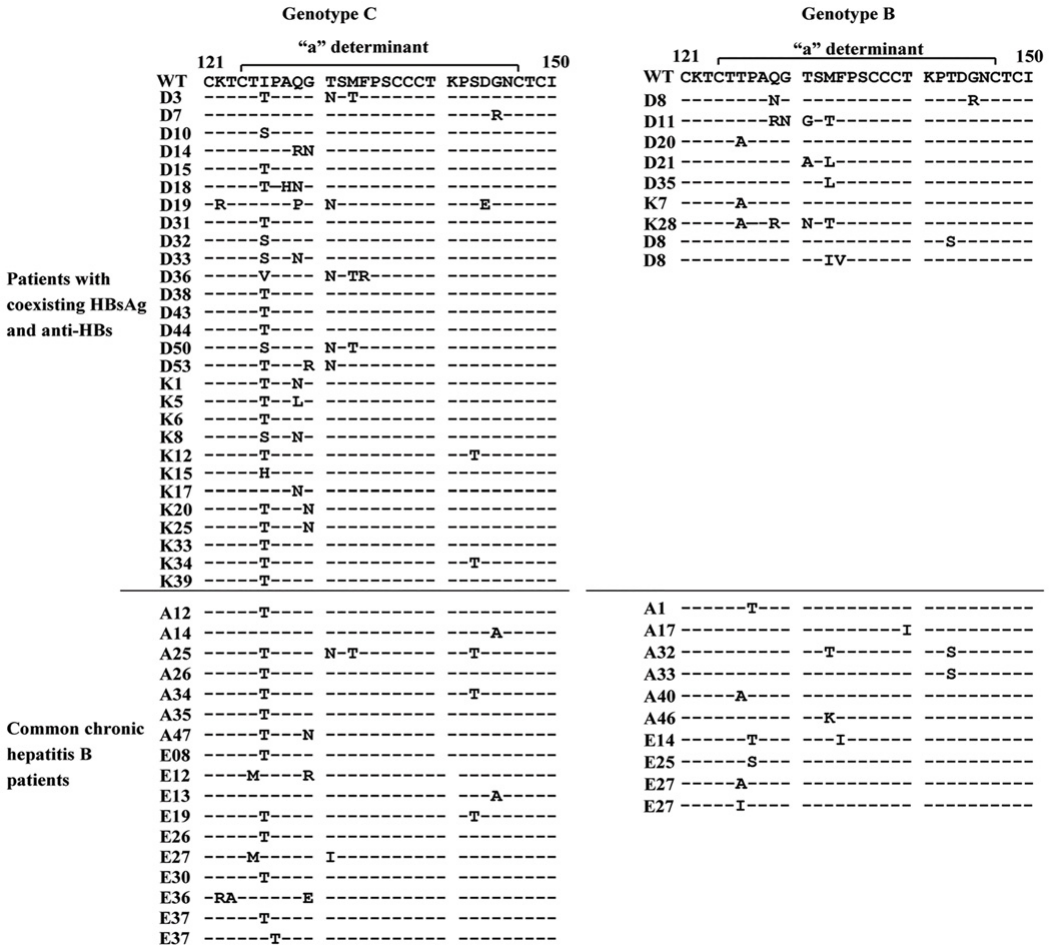
Point mutations in the *a* determinant. The amino acid sequences derived from patients with mutation in the *a* determinant were compared to reference wild-type sequences from the same genotype.

It is reported that the coexistence of HBsAg and anti-HBs was associated with an increase of point mutations in the *a* determinant region (amino acids 124–147) of HBsAg [9,11]. Thus, here we focused on the difference of *a* determinant mutations between the two groups. As shown in Table 3, point mutations in the *a* determinant were detected in 37 (57.8%) of 64 patients from the research group, and the mutations were detected in 27 (38.6%) of 70 patients of control group (P < 0.05). However, in the patients with genotype B, the rate of point mutations in the *a* determinant was 39.1% (9/23) in the patients from the research group, and the rate was 41.7% (10/24) in the control group (P = 0.859). Of the patients with genotype C, the rate was 68.3% (28/41) in the patients from the research group, while the rate was 36.9% (17/46) in the control group (P < 0.01). The point mutations at amino acid 126, 129, 131, and 133 were frequently identified within the *a* determinant in patients from the research group (Table 3, Fig 2). However, the G145 R/A mutation, which is able to evade neutralizing anti-HBs and infect vaccinated individuals [18], was similar in the two groups (2/64 vs 2/70).

**Table 3:** Comparison the amino acid mutations of *a* determinant between the patients with coexisting HBsAg and anti-HBs and controls.

	Patients with coexisting HBsAg and anti-HBs (n = 64)	Control group (n = 70)	P value
**Rate of mutations**			
** Genotype B**	9/23 (39.1%)	10/24 (41.7%)	0.859
** Genotype C**	28/41 (68.3%)	17/46 (37.0%)	0.004
**Total**	37/64 (57.8%)	27/70 (38.6%)	0.026
**Point mutations**			
** 126 T/I**	26/64 (40.6%)	14/70 (20%)	0.009
** 129 Q**	11/64 (17.2%)	0/70 (0%)	< 0.01
** 131 T**	8/64 (12.5%)	2/70 (2.86%)	0.034
** 133 M**	8/64 (12.5%)	3/70 (4.29%)	0.084

### Follow-up analysis

A positive anti-HBs generally indicates protective immunity to HBV. Thus, it will be interesting to follow up these patients to clarify whether the presence of anti-HBs would favor the clinical course. We followed 14 patients with positive HBsAg and anti-HBs for an average of 15.5 months (range 4–33 months), and none of them received any antiviral therapy. During the follow-up period, there was no significant change in the levels of HBsAg, anti-HBs, HBV DNA and ALT as well as positive rate of HBeAg (P > 0.05) (Table 4).

**Table 4:** Comparison the virological characteristics of HBV infection during the follow-up.

	First visiting (n = 14)	Follow-up (n = 14)	P value
**HBsAg (IU/ml)**	99.43 (32.16, 249.74)	162.16 (10.42, 490)	0.109
**Positive rate of HBeAg**	7/14 (50%)	6/14 (42.9)	0.705
**HBV DNA (Log10 IU/ml)**	4.60 ± 1.76	3.93 ± 1.66	0.144
**ALT (U/L)**	47.1 (30.6, 89.4)	59.3 (25.9, 74.6)	0.875
**Anti-HBs (mIU/ml)**	27.08 (18.06, 70.10)	35.7 (10.34, 76.43)	0.594

Of the 14 patients, 12 had the same amino acid sequences during the follow-up period, while the patients DS-22 and DS-32 were observed to have different amino acid sequences in the MHR of HBsAg at the end of their follow-up time. After 4 months of follow-up, 2 amino acid substitution mutations (I126T and S143T) in the ‘‘a” determinant were identified in the sample from patient DS-22 compared to the patient’s baseline sequences. After 27 months of follow-up, the sample from patient DS-32 had a 4-amino acid insertion between s118 and s119 in the S gene, and the insertion is a repeat sequence of preceding 4 amino acids. Meanwhile the sequence also had one amino acid substitution mutations (G145R) in the ‘‘a” determinant.

## Discussion

In the present study, we studied the clinical and virological characteristics of patients with coexisting HBsAg and anti-HBs in China. We found that, of 13080 HBV-infected patients, 3.33% were simultaneous positive for anti-HBs, which is comparable to the reported 2.43–3.6% in China and France [7,9,13], but somewhat lower than that reported rate (6.4–8.9%) by Jang in South Korea and Lada in France [11,19]. Noticeably, the mean age of the patients with coexisting HBsAg and anti-HBs was 56.8 years old, and 68.75% individuals were > 50 years old in our study. We observed that, compared with common hepatitis B patients, the patients with positive anti-HBs had lower HBsAg and HBV DNA levels, higher amino acid variability in the HBsAg and RT region.

Anti-HBs is the major neutralizing antibody protecting against infection and reflects immunity against HBV infection [18]. In our study, the patients with positive anti-HBs had lower HBsAg and HBV DNA levels, which indicate that anti-HBs could partly neutralize HBsAg and clear HBV particles in the circulation. However, during the follow-up period, most patients with coexisting HBsAg and anti-HBs had the same pattern of serological markers and constant levels of HBV DNA and HBsAg, which indicate that the anti-HBs in chronic HBV-infected patients may not fully neutralize HBV.

Previous reports suggested that the coexistence of HBsAg and anti-HBs might reflect the severity of liver disease and the active replication or reactivation of virus [8,19,20]. However, in our study the HBsAg, HBV DNA and ALT levels in patients with positive anti-HBs were lower than that in common hepatitis B patients. Moreover, more than 50% patients with coexisting HBsAg and anti-HBs were asymptomatic carriers. Meanwhile, during the follow-up period, the levels of HBsAg, anti-HBs, HBV DNA, and positive rate of HBeAg as well as ALT had no significant change. Therefore, our study showed no evidence to indicate that coexistence of HBsAg and anti-HBs may be associated with the severity of chronic liver disease.

Nowadays, selection of the immune escape mutations is considered to be the main reason of coexistence of HBsAg and anti-HBs [5,6,21]. The mutations include the deletions in the pre-S gene and the mutation in the regions of S protein of HBV, especially in the *a* determinant [5,6,22,23]. However, the results in different studies are not entirely comparable. In our study, HBV pre-S deletion mutations are less likely to be related to the coexistence of HBsAg and anti-HBs since the two groups had similar rates of pre-S deletion mutations. Our data are not consistent with other results, in which HBV pre S deletion mutations were related to the coexistence of HBsAg and anti-HBs [22]. In addition, compared to the control group, the patients with coexisting HBsAg and anti-HBs had higher amino acid variability within the C-terminal region and the MHR, but not within the N-terminal region. The results are also not consistent with previous reports, in which the amino acid exchange mostly occurred within the MHR and N-terminal region [7,11]. The different results in different studies may be associated with the selection of patients and sampling errors, since other studies were limited by their small sample size [7,11,22].

In our study the most frequent mutations were located at the first loop of *a* determinant, which may be associated with alteration of HBsAg antigenicity, and the results are comparable to the previous studies [6,12,13]. While it seems that the G145R/A mutation, which is considered the most frequently mutation in patients with coexisting HBsAg and anti-HBs [9,11], is not common in this study. This result is also comparable to other reports in China [7,12,22]. In addition, in the patients with coexistence of HBsAg and anti-HBs, the patients with HBV genotype C have more point mutations in the *a* determinant than that with genotype B in our study. While for the patients with genotype B, there was no significant difference in the point mutations within the *a* determinant between the research group and control group. The similar results were also reported by Ding in China [7].

It is reported that the immune pressure in chronic HBV-infected patients may lead to escape mutants [6,24]. In our follow-up study, the calculated mean number of nucleotide substitutions/site/year (6.9 × 10^−4^) was much higher than the mutation rate occurred in chronic HBV-infected patients (1.5 × 10^−5^ to 7.9 × 10^−5^) [25]. Notably, all the mutations occurred during the follow-up period were located in the MHR of HBsAg, indicating that mutation in HBsAg MHR is associated with the presence of anti-HBs.

There are several limitations in the present study. A major limitation in our study is that the small size of the study subjects and the smaller one (only 14 cases) at follow-up investigation to describe the clinical characteristics. The reason was that most of the patients were from other cities or rural areas, and it is hard to take long-term follow-up of these patients. Thus, we just analyzed the short-term impact of anti-HBs on the patients. Second, we only observed the results of HBV DNA, ALT and HBsAg levels, but did not observe the full disease picture, such as liver cirrhosis, HCC, and other clinical outcomes. Third, we just sequenced the PCR products directly and did not clone the sequences, probably leading to missing the minor mutations.

In summary, the patients with coexisting HBsAg and anti-HBs may have higher variability within the C-terminal region and the MHR of HBsAg. The presence of anti-HBs in chronic HBV-infected patients may be associated with mutations in the S regions of HBV, especially in the *a* determinant of HBV genotype C. Since the patients with coexisting HBsAg and anti-HBs have lower HBsAg and HBV DNA levels, it merits further study to determine how the coexistence of HBsAg and anti-HBs influences on the clinical course and severity of chronic hepatitis B.

## Supporting Information

S1 Table General characteristics of the study population. (XLSX)

S1 Fig Phylogenetic analysis of S region of HBV. Phylogenetic tree of the S gene sequences from chronic HBV-infected patients with or without anti-HBs positive in this study and sequences recovered from GenBank. Sequences retrieved from GenBank are denoted by their accession numbers and genotype.
(TIF)
