# Supplementary figures and images for: Clinical and Virological Characteristics of Chronic Hepatitis B Patients with Coexistence of HBsAg and Anti-HBs
Source: PLoS One. 2016 Jan 11;11(1):e0146980. doi: 10.1371/journal.pone.0146980 (PMC4709170; doi:10.1371/journal.pone.0146980)

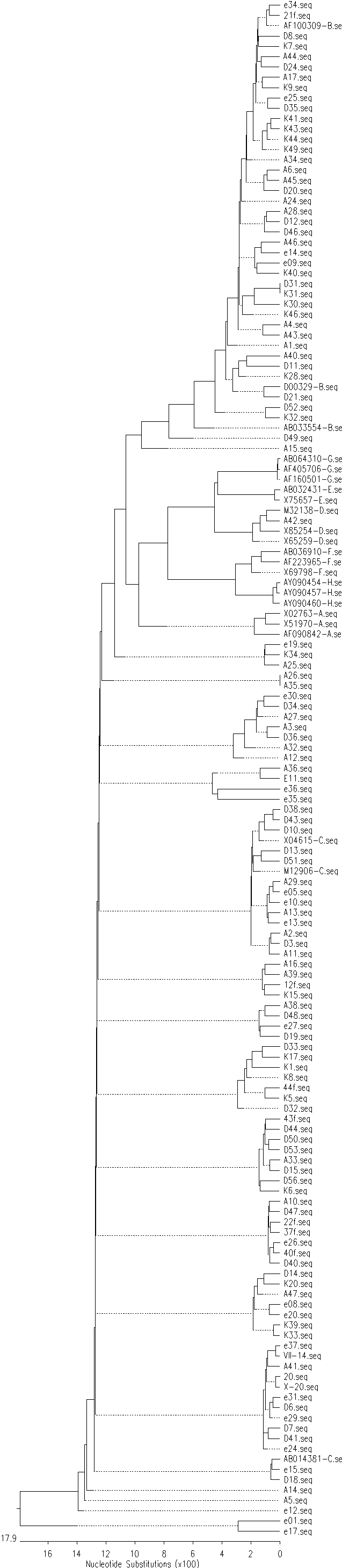

Supplement: S1 Fig — Phylogenetic tree of the S gene sequences from chronic HBV-infected patients with or without anti-HBs positive in this study and sequences recovered from GenBank. Sequences retrieved from GenBank are denoted by their accession numbers and genotype. (TIF) [file pone.0146980.s002.tif]
